# Altered inflammatory responsiveness in serotonin transporter mutant rats

**DOI:** 10.1186/1742-2094-10-116

**Published:** 2013-09-19

**Authors:** Flavia Macchi, Judith R Homberg, Francesca Calabrese, Claudia Zecchillo, Giorgio Racagni, Marco A Riva, Raffaella Molteni

**Affiliations:** 1 Dipartimento di Scienze Farmacologiche e Biomolecolari, Università degli Studi di Milano, Milan, Italy; 2 Department of Clinical and Molecular Biomedicine, Section of Pharmacology and Biochemistry, University of Catania, Catania, Italy; 3 Donders Institute for Brain Cognition and Behaviour, Radboud University Nijmegen Medical Centre, Nijmegen, The Netherlands; 4 Center of Excellence on Neurodegenerative Diseases, Università degli Studi di Milano, Milan, Italy

**Keywords:** Inflammation, Lipopolysaccharide (LPS), Cytokines, Microglia, Animal model

## Abstract

**Background:**

Growing evidence suggests that alterations of the inflammatory/immune system contribute to the pathogenesis of depression. Indeed, depressed patients exhibit increased levels of inflammatory markers in both the periphery and the brain, and high comorbidity exists between major depression and diseases associated with inflammatory alterations. In order to characterize the link between depression and inflammation, we aimed to investigate whether an altered inflammatory system is present in a genetic model of vulnerability for depression, namely rats with partial or total deletion of the serotonin transporter (SERT) gene.

**Methods:**

Wild-type, heterozygous and homozygous SERT rats were analyzed under basal condition or following a challenge with an acute injection of lipopolysaccharide (LPS) and killed 24 h or 5 days later.

**Results:**

We found that SERT mutant rats show altered cytokine expression in the dorsal and ventral hippocampus at basal conditions, and they also display an exacerbated cytokine response to the LPS challenge. Moreover, mutant rats exhibit differences in the expression of markers for microglia activation.

**Conclusion:**

Based on these data, we suggest that basal or functional alterations of immune/inflammatory systems might contribute to the phenotype of SERT rats and to their heightened susceptibility to depressive-like behavior.

## Background

Major depressive disorder (MDD) is a leading cause of disability in the world supposedly originating from the interaction between a background of genetic vulnerability and adverse environmental factors. Compelling evidence also suggests that inflammation may contribute to specific dysfunctions associated with depression [1,2]. Accordingly, depression shows elevated comorbidity with immune-related diseases such as cancer, cardiovascular and neurodegenerative diseases that are characterized by the presence of inflammatory alterations [3,4]. In addition, depressed patients exhibit all the cardinal features of inflammation [5]. Indeed, elevated blood levels of the proinflammatory cytokines, including interleukin (IL)-1β, IL-6 and tumor necrosis factor (TNF)-α, are commonly found in depressive subjects [6-9]. It has also been shown that depressed suicidal attempters display elevated levels of IL-6 in the cerebrospinal fluid [10]. Moreover, it has been demonstrated that IL-6 is related to the stress-induced development of depression-like behaviors in mice [11].

Furthermore, the endotoxin lipopolysaccharide (LPS), a proinflammatory agent, can trigger the development of depressive symptoms in humans as well as depressive-related behavior in rodents [1,12-14]. In line with this, patients treated with interferons and interleukins often develop depression [15]. This evidence suggests that immune-inflammatory signals may represent an ‘environmental’ condition relevant for the etiology of mood disorders, which may also unmask a latent genetic vulnerability.

In this respect, one of the most extensively investigated susceptibility genes in depression encodes for the serotonin transporter (5-HTT or SERT), which is responsible for the re-uptake of serotonin into the presynaptic terminal [16]. The 5-HTT gene exists in two major allelic variants, a long (L) form and a short (S) form. It has been demonstrated that the S variant leads to reduced expression of the transporter [17] and might enhance the sensitivity to the pro-depressive effects of stressful life events in rhesus monkeys [18] as well as in humans [19,20]. Furthermore, SERT knockout mice [21-23], as well as SERT mutant rats [24], show depression and anxiety-related behaviors, as well as impaired neuronal plasticity [25-27], supporting the possibility that altered function of SERT may be associated with increased risk of mood disorders [28]. Interestingly, it has been recently shown that the short variant of SERT is also associated with higher risk of developing depression in patients following INF-α treatment [29].

Based on these observations, the purpose of the present study was twofold. First, we established whether partial or total deletion of the SERT gene is associated with altered expression of inflammatory markers in basal conditions. Next, in order to establish whether genetic susceptibility to depression is associated with significant changes in the inflammatory/immune system, we investigated whether SERT heterozygous rats show altered responsiveness to an inflammatory challenge with LPS. We chose SERT^+/−^ animals since the partial SERT deletion better mimics the condition of humans carrying the short variant of the 5-HTTLPR, which has been associated with enhanced susceptibility to environmental adversities [30]. The higher validity of SERT heterozygous models has already been discussed [31,32], and the behavioral vulnerability of SERT heterozygous animals has been previously demonstrated for mice [21,33].

## Methods

General reagents were purchased from Sigma-Aldrich (Milan, Italy), and molecular biology reagents were obtained from Applied Biosystem Italia (Monza, Italy), Eurofins MWG-Operon (Ebersberg, Germany) and Bio-Rad Laboratories S.r.l. (Segrate, Italy). Lipopolysaccharide (from *Escherichia coli* 026:B6 ≥10,000 eu/mg) was purchased from Sigma-Aldrich (code L8274).

### Animals and experimental paradigm

Serotonin transporter knockout rats (Slc6a4^1Hubr^) were generated in a Wistar background by N-ethyl-N-nitrosurea (ENU)-induced mutagenesis [34]. Experimental animals were derived from crossing heterozygous SERT knockout rats that were out crossed for at least ten generations with wild-type Wistar rats obtained from Harlan Laboratories. All subjects were bred and reared in the Central Animal Laboratory of the University of Nijmegen. After weaning at the age of 21 days, ear cuts were taken for genotyping. In all experiments, adult male SERT^+/+^(WT), SERT^+/−^ and SERT^−/−^ rats were used. Animals were housed in groups of four per cage under standard conditions (12-h light/dark cycle with food and water *ad libitum*) and were exposed to daily handling for 1 week before any treatment.

Rats were randomly divided into two experimental groups: control (39 rats) and treated animals (40 rats); the first group received saline, whereas the second one was treated with a single injection of lipopolysaccharide (250 μg/Kg, i.p.). They were killed 1 or 5 days later. Our analyses were carried out in the ventral and dorsal hippocampus, which were rapidly dissected, frozen on dry ice and stored at −80°C for molecular analyses.

All experiments were approved by the Committee for Animal Experiments of the Radboud University Nijmegen Medical Centre, Nijmegen, The Netherlands, and all efforts were made to minimize animal suffering and to reduce the number of animals used in accordance with the Guidelines laid down by the European Communities Council Directive of 24 November 1986 (86/609/EEC).

### Protein analysis of plasma inflammatory mediators

Samples of blood from each rat were collected in heparinized tubes. Plasma was separated by centrifugation (5,000 rpm for 10 min at 4°C), and interleukin (IL)-6, tumor necrosis factor (TNF)-α, cytokine-induced neutrophil chemoattractant (CINC)-1, CINC-3 and macrophage inflammatory protein (MIP)-1α protein levels were quantified using a Rat Cytokine Array kit (R&D Systems, Inc.) according to the manufacturer’s instructions. Briefly, plasma was diluted and mixed with a cocktail of biotinylated detection antibodies. The sample/antibody mixture was then incubated with the rat cytokine array membrane where capture and control antibodies have been spotted. Any cytokine/detection antibody complex present was bound by its cognate immobilized capture antibody on the membrane. Following a wash to remove unbound material, streptavidin-HRP and chemiluminescent detection reagents were applied and a signal was produced at each spot corresponding to the amount of cytokine bound. Protein levels were calculated by measuring the optical density of the autoradiographic bands using Quantity One software (Bio-Rad Laboratories). To ensure that autoradiographic bands were in the linear range of intensity, different exposure times were used.

### RNA preparation and gene expression analyses

For gene expression analysis, total RNA was isolated from the different brain regions by single-step guanidinium isothiocyanate/phenol extraction using the PureZol RNA isolation reagent (Bio-Rad Laboratories S.r.l.; Segrate, Italy) according to the manufacturer’s instructions and quantified by spectrophotometric analysis. The samples were then processed for real-time polymerase chain reaction (PCR) as previously reported [35] to assess levels of interleukin 1β (IL-1β) interleukin-6 (IL-6), integrin alpha M (cluster of differentiation molecule CD11b), chemokine (C-X3-C motif) ligand 1 (CX3CL1; fractalkine) and integrin-associated protein (CD47).

Briefly, an aliquot of each sample was treated with DNase to avoid DNA contamination and subsequently analyzed by the TaqMan qRT-PCR instrument (CFX384 real-time system, Bio-Rad Laboratories S.r.l.) using the iScript one-step RT-PCR kit for probes (Bio-Rad Laboratories S.r.l.). Samples were run in 384-well format in triplicate as multiplexed reactions with a normalizing internal control (β-actin). Thermal cycling was initiated with incubation at 50°C for 10 min (RNA retrotranscription) and then at 95°C for 5 min (TaqMan polymerase activation). After this initial step, 39 cycles of PCR were performed. Each PCR cycle consisted of heating the samples at 95°C for 10 s to enable the melting process, and then for 30 s at 60°C for the annealing and extension reactions. A comparative cycle threshold (Ct) method was used to calculate the relative target gene expression. Probe and primer sequences used were purchased from Applied Biosystem Italia and Eurofins MWG-Operon.

### Statistical analyses

The effect of the genotype on gene expression was analyzed with a one-way analysis of variance (ANOVA), followed by Fisher’s protected least significant difference (Fisher PLSD), whereas the effect of the LPS was analyzed with a two-way ANOVA with genotype (WT vs. SERT^+/−^ rats) and treatment (Sal vs. LPS) as independent factors and mRNA levels as dependent variable. When appropriate, further differences were analyzed by single contrast post hoc test (SCPHT). Significance for all tests was assumed at *P* < 0.05. Data are presented as means ± standard error (SEM). For graphic clarity, results are presented as mean percent of WT rats (basal effect analysis) and WT or SERT^+/−^ rats treated with saline (LPS effect analysis).

## Results

We first analyzed different markers of the immune system at the peripheral level of SERT^+/−^ and SERT^−/−^ animals. Specifically, we measured the protein level of two cytokines, IL-6 and TNF-α and CINC-1, CINC-3 and MIP-1α, which belong to the family of chemokines. As shown in Table 1, all these markers were strongly upregulated in the plasma of SERT^−/−^ rats, whereas we did not find any change in SERT heterozygous animals.

**Table 1:** Protein analysis of inflammatory mediators in the plasma of SERT mutant rats

***Gene***	**SERT +/−**	**SERT −/−**
CINC-1	=	+
CINC-3	=	+++
MIP-1α	=	+++
IL-6	=	++
TNF-α	=	+

The table shows the changes of interleukin 6 (IL-6), tumor necrosis factors (TNF-α), cytokine-induced neutrophil chemoattractant 1 (CINC-1), cytokine-induced neutrophil chemoattractant 3 (CINC-3) and macrophage inflammatory protein (MIP-1α) found in SERT mutant as compared to wild-type animals. =, no change; +, 5- to 10-fold increase; ++ 20- to 30-fold increase; +++ more than 30-fold increase.

On these bases, we decided to analyze the expression levels of inflammatory markers in the hippocampus of mutant rats under resting conditions or following an acute challenge with LPS. We chose to investigate this brain area because it is highly relevant for depression and it is also vulnerable to environmental challenges [36-38]. Moreover, we decided to compare the ventral and dorsal part of the hippocampus since the two subregions subserve different functions. In particular, the dorsal part (DH) has been linked to cognition, whereas the ventral portion (VH) has been associated with emotion and stress responses [39].

### IL-1β expression

**Figure 1:**
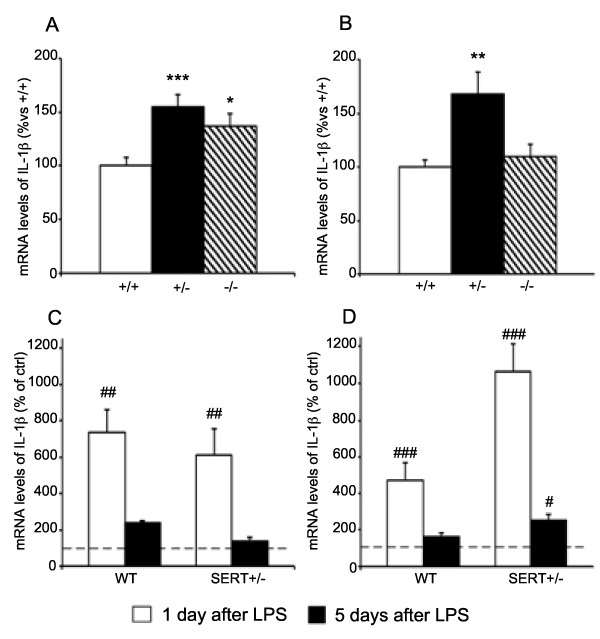
**Analysis of interleukin (IL)-1β gene expression in the hippocampus of SERT mutant rats.** Basal expression of IL-1β mRNA levels were measured in dorsal **(A)** and ventral **(B)** hippocampus of mutant rats. The data, expressed as percentage of SERT^+/+^ (wild-type) animals (set at 100%), are the mean ± SEM of at least five independent determinations. *P < 0.05, **P < 0.01 and ***P < 0.001 vs. SERT^+/+^ (one-way ANOVA with SCPHT). The expression of IL-1β was also investigated in dorsal **(C)** and ventral **(D)** hippocampus following an acute LPS challenge, the animals being killed 1 or 5 days after the immune challenge. The data are expressed as percentage of the respective saline-injected counterpart (set at 100%, *dashed line*) and represent the mean ± SEM of at least five independent determinations. #*P* < 0.05, ##*P* < 0.01, ###*P* < 0.001 vs. control (one-way ANOVA with SCPHT).

We first measured IL-1β mRNA and, as shown in Figure 1, we found that under basal conditions the expression of IL-1β was significantly increased in the dorsal hippocampus (A) of SERT^+/−^ and SERT^−/−^ animals (+55%, *P* < 0.001; +37%, *P* < 0.05 respectively), whereas, in the ventral hippocampus, (B) the cytokine mRNA levels were increased only in rats with partial deletion of the gene (+68%, *P* < 0.01). Next, we investigated the responsiveness of SERT^+/−^ rats to a challenge with the proinflammatory agent LPS. The analyses were carried out only in SERT^+/−^ rats, which more closely mimic the genetic vulnerability associated with patients carrying the S variant of the 5-HTT gene. In the dorsal hippocampus (Figure 1C), we found that LPS produced similar changes in both genotypes. Indeed, the mRNA levels for IL-1β were significantly increased 1 day after LPS injection in WT (+634%, *P* < 0.01) as well as in SERT^+/−^ rats (+513%, *P* < 0.05), but not after 5 days when the cytokine expression levels had returned to control levels. Conversely, in the ventral hippocampus (Figure 1D), LPS injection produced larger changes in SERT^+/−^ rats since IL-1β upregulation at 24 h was more pronounced in mutant animals (+964% vs. +370% in WT rats) and also persisted up to 5 days after the proinflammatory challenge (+156%, *P* < 0.05) when the cytokine expression had returned to control levels in wild-type rats.

### IL-6 expression

**Figure 2:**
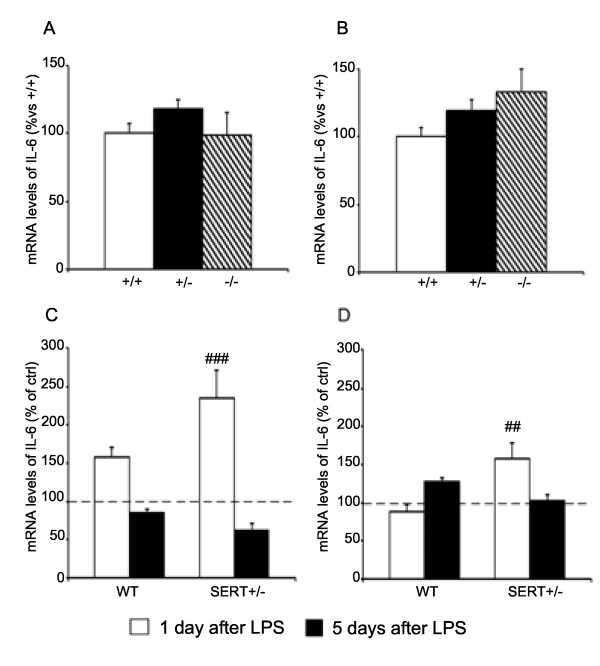
**Analysis of interleukin (IL)-6 gene expression in the hippocampus of SERT mutant rats.** Basal interleukin (IL)-6 gene expression is not altered in dorsal **(A)** or ventral **(B)** hippocampus of mutant rats. The data, expressed as percentage of SERT^+/+^ (wild-type) animals (set at 100%), are the mean ± SEM of at least five independent determinations. The expression of IL-6 is upregulated in both dorsal **(C)** and ventral **(D)** hippocampus only in SERT^+/−^ animals 1 day after an lipopolysaccharide (LPS) challenge. The data are expressed as percentage of the respective saline-injected counterpart (set at 100%, dashed line) and represent the mean ± SEM of at least five independent determinations. ##*P* < 0.01, ###*P* < 0.001 vs. control (one-way ANOVA with SCPHT).

Unlike what we observed for IL-1β, the mRNA levels of IL-6 (Figure 2) were not significantly altered under basal conditions in the dorsal (A) or ventral hippocampus (B) of SERT mutant rats. However, we found that the SERT genotype had a significant effect on the modulation of IL-6 gene expression after LPS challenge. In the dorsal hippocampus (Figure 2C), 24 h after LPS injection, IL-6 mRNA levels were significantly increased in SERT^+/−^ (+136%, *P* < 0.001) but not wild-type animals (+58%, *P* = 0.067), whereas the cytokine expression was back to control levels in both genotypes 5 days post-LPS. A similar profile was found in the ventral hippocampus (Figure 2D), since IL-6 expression was upregulated 24 h post LPS administration in SERT^+/−^ (+58%, *P* < 0.01) but not in WT rats (−11%, *P* = 0.565). Also in this brain region, no significant changes of IL-6 mRNA levels were found 5 days after LPS challenge in both genotypes.

### CD11b expression

**Figure 3:**
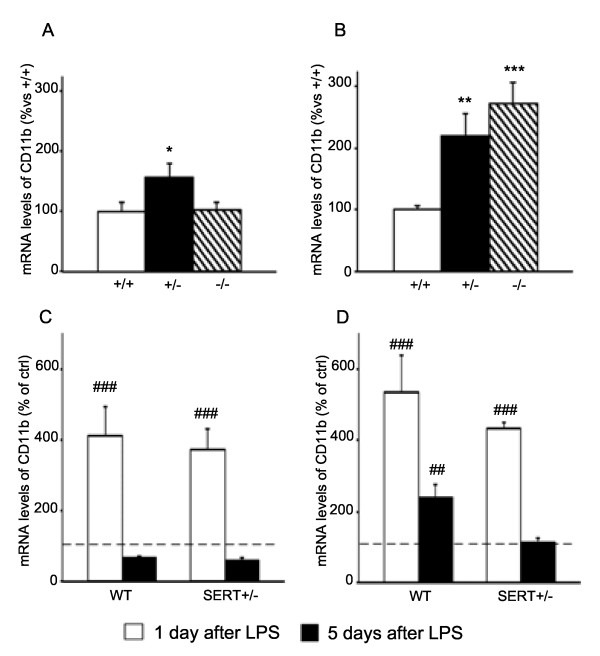
**Analysis of integrin alpha M (CD11b) gene expression in the hippocampus of SERT mutant rats.** Basal CD11b gene expression is altered in dorsal **(A)** and ventral **(B)** hippocampus of mutant rats. The data, expressed as percentage of SERT^+/+^ animals (set at 100%), are the mean ± SEM of at least five independent determinations. *P < 0.05, **P < 0.01 and ***P < 0.001 vs. SERT^+/+^ (one-way ANOVA with SCPHT). The expression of CD11b is upregulated in dorsal **(C)** and ventral **(D)** hippocampus of WT or SERT^+/−^ animals 1 or 5 days after an LPS challenge. The data are expressed as percentage of the respective saline-injected counterpart (set at 100%, *dashed line*) and represent the mean ± SEM of at least five independent determinations. ##*P* < 0.01, ###*P* < 0.001 vs. control (one-way ANOVA with SCPHT).

Besides cytokine production, another important aspect of the inflammatory response is microglia activation [40]. In order to evaluate if the basal activity of microglia was altered as a consequence of SERT gene deletion, we assessed the expression of CD11b, a marker for this cellular phenotype [41]. As shown in Figure 3A, we found that the basal expression of CD11b was significantly higher in dorsal hippocampus of SERT^+/−^ (+56%, *P* < 0.05) but not in SERT^−/−^ rats. A similar change was also observed in the ventral hippocampus (Figure 3B) where the expression of the microglial marker was significantly upregulated under basal conditions in SERT^+/−^ (+120%, *P* < 0.01) as well as in SERT^−/−^ rats (+173%, *P* < 0.001).

Next, we examined CD11b mRNA levels following the acute LPS challenge. In the dorsal hippocampus (Figure 3C), the mRNA levels of CD11b were significantly increased 1 day after LPS treatment in WT (+312%, *P* < 0.001) as well as in SERT^+/−^ rats (+274%, *P* < 0.001), while its expression returned to basal levels in both genotypes 5 days post-LPS. In the ventral hippocampus (Figure 3D), we found a significant increase of CD11b mRNA levels 24 h after LPS injection in WT (+436%, *P* < 0.001) and SERT^+/−^ rats (+332%, *P* < 0.001). However, 5 days after LPS challenge the mRNA levels of CD11b were upregulated in WT rats (140%, *P* < 0.001), but they had returned to baseline in SERT^+/−^ rats.

### CX3CL1 and CD47 expression

In order to gain further insight into the changes of microglial function, we investigated the glia-neuron cross-talk. Indeed, microglial function may be controlled through signals that keep it in a resting state or that may favor its activation [42]. In this respect, the interactions between fractalkine (CX3CL1) and CD47 with their receptors play a crucial role in maintaining microglia in a resting state [43]. Therefore, we analyzed the expression of CX3CL1 and CD47 in the dorsal and ventral hippocampus of SERT^+/−^ and wild-type animals.

**Figure 4:**
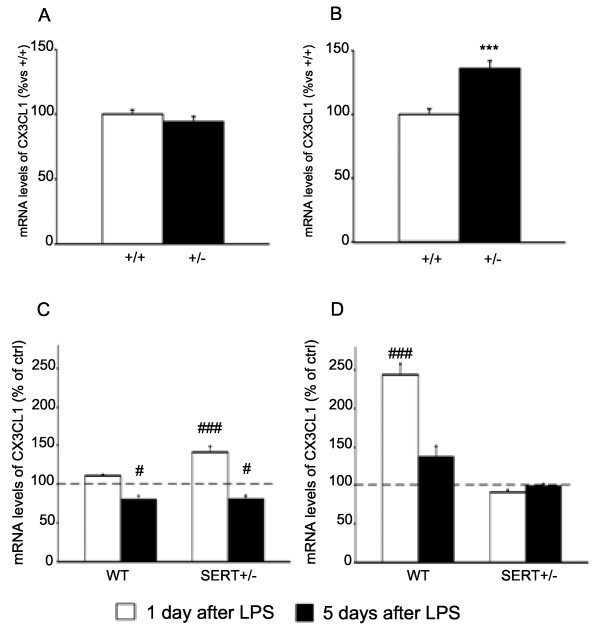
**Analysis of chemokine ligand 1 (CX3CL1) gene expression in the hippocampus of SERT mutant rats.** Basal CX3CL1 gene expression is altered in the ventral hippocampus of mutant rats **(B)** but not in the dorsal hippocampus **(A)**. The data, expressed as percentage of SERT^+/+^ animals (set at 100%), are the mean ± SEM of at least five independent determinations. ***P < 0.001 vs. SERT^+/+^ (one-way ANOVA with SCPHT). The expression of CX3CL1 is modulated in dorsal **(C)** and ventral **(D)** hippocampus of WT or SERT^+/−^ animals 1 or 5 days after an LPS challenge. The data are expressed as percentage of the respective saline-injected counterpart (set at 100%, *dashed line*) and represent the mean ± SEM of at least five independent determinations. #*P* < 0.5, ###*P* < 0.001 vs. control (one-way ANOVA with SCPHT).

As shown in Figure 4A, the expression of CX3CL1 was not altered in the dorsal hippocampus of SERT^+/−^ rats under basal conditions, while its mRNA levels were increased in the ventral subregion of mutant rats (+36%, *P* < 0.001).

When considering the inflammatory challenge, CX3CL1 mRNA levels were not altered in the dorsal hippocampus of WT rats 24 h after LPS treatment (Figure 4C), while they were significantly increased in SERT^+/−^ animals (+42%, *P* < 0.001). Conversely, 5 days after the LPS challenge, the expression of CX3CL1 was significantly reduced in both genotypes (WT: -19%, *P* < 0.05; SERT+/−: -20%, *P* < 0.05). In the ventral hippocampus (Figure 4D), the mRNA levels of CX3CL1 were upregulated 1 day after LPS injection in WT rats (+144%, *P* < 0.001), but not in SERT^+/−^ animals, whereas no significant changes were observed 5 days post-LPS challenge.

**Figure 5:**
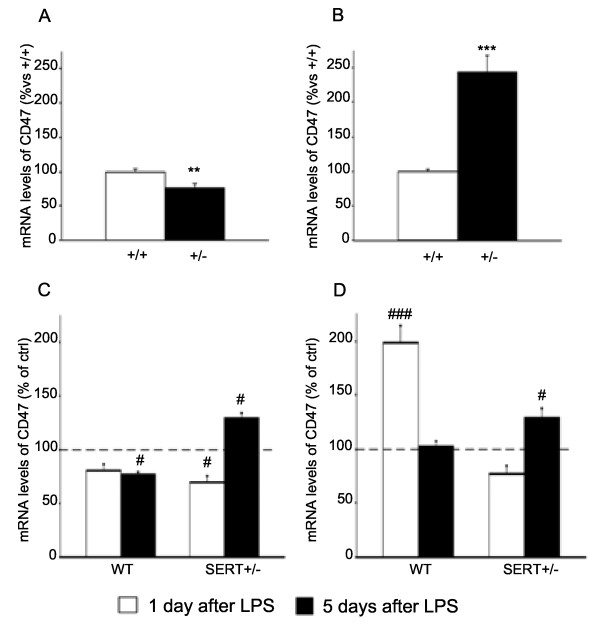
**Analysis of integrin-associated protein (CD47) gene expression in the hippocampus of SERT mutant rats.** Basal CD47 mRNA levels are decreased in the dorsal hippocampus **(A)** of SERT^+/−^ rats, while they are increased in the ventral hippocampus **(B)**. The data, expressed as percentage of SERT^+/+^ animals (set at 100%), are the mean ± SEM of at least five independent determinations. **P < 0.01 and ***P < 0.001 vs. SERT^+/+^ (one-way ANOVA with SCPHT). The expression of CD47 is modulated in dorsal **(C)** and ventral **(D)** hippocampus of WT or SERT^+/−^ animals 1 or 5 days after an LPS challenge. The data are expressed as percentage of the respective saline-injected counterpart (set at 100%, *dashed line*) and represent the mean ± SEM of at least five independent determinations. #*P* < 0.05, ###*P* < 0.001 vs. control (one-way ANOVA with SCPHT).

Conversely, as shown in Figure 5, basal expression of CD47 was reduced in the dorsal hippocampus of SERT^+/−^ rats (−34%, *P* < 0.01), while it was increased in the ventral hippocampus (+144%, *P* < 0.001). Following LPS challenge, the expression of CD47 was not altered in the dorsal hippocampus of WT rats 24 h post-injection, and it was reduced 5 days after (−23%, *P* < 0.05), (Figure 5C). Conversely, the expression of CD47 was decreased at 24 h post-LPS in SERT^+/−^ animals (−30%, *P* < 0.05), but was significantly upregulated 5 days post-LPS injection (+30%, *P* < 0.05). In the ventral hippocampus (Figure 5D), the mRNA levels of CD47 were upregulated 1 day after LPS administration in WT rats (+99%, *P* < 0.001) but not in SERT^+/−^ animals. On the contrary, 5 days after the inflammatory challenge the expression of the microglial marker had returned to control values in WT rats, while being significantly increased in SERT mutant rats (+29%, *P* < 0.05).

## Discussion

Our results suggest that genetic deletion of the serotonin transporter in rats is associated with alterations of immune/inflammatory players, such as proinflammatory cytokines and markers of microglia activation, under basal conditions or following an immune challenge. These results support the idea of a close and reciprocal modulation between a gene strongly associated with depression and systems involved in the immune response, in line with the idea that inflammation represents an important environmental factor for depression susceptibility [1,44,45]. Indeed, we demonstrate that animals with partial or total deletion of the SERT gene have, under basal conditions, enhanced levels of circulating immune proteins as well as increased expression of IL-1β in dorsal and ventral hippocampus. In this respect, it is interesting to notice that major changes occurred in heterozygous animals, which mimic more closely the human situation of individuals carrying the short (S) variant of the SERT gene that is associated with enhanced vulnerability to depressive disorders [19,20]. Besides this, Su and colleagues have demonstrated that in S-carrier subjects depressive symptoms are associated with elevated plasma levels of IL-6 [46]. Moreover, healthy SS carriers of the serotonin transporter show, when compared to LL carriers, a proinflammatory phenotype, measured as the ratio between IL-6 and IL-10, under resting conditions as well as following an acute stress [47]. These data are in good agreement with the results of our study, also in respect to the responsiveness to an immune challenge. In particular, while cytokine mRNA levels are similarly increased in the dorsal hippocampus of all genotypes, the response to LPS was exacerbated in the ventral hippocampus of SERT^+/−^ rats, where the cytokine upregulation was not only quantitatively larger but also lasted up to 5 days after the inflammatory challenge. The anatomical specificity is in line with the potential role of these changes for depression considering that the ventral subregion of the hippocampus is primarily involved in emotional responses and stress regulation [48].

One key element of the inflammatory response is the activation and modulation of microglia [40]. These cerebral immune cells are normally present in the healthy brain where they actively survey the system and may rapidly respond to any microenvironment alteration [49]. To substantiate the phenotype of SERT mutant rats, we found increased mRNA levels of CD11b in the ventral and dorsal hippocampus. In particular, SERT^+/−^ rats displayed more obvious alterations, suggesting once again that heterozygous rats are more sensitive to an LPS challenge. Indeed, we observed a genotype-dependent increase in CD11b: after 24 h from LPS administration there was no difference, whereas after 5 days microglia was still activated in wild-type but not in SERT^+/−^ animals. The interpretation of these results is not straightforward, since the mechanisms underlying microglia activation and its functional consequences are not completely understood. Indeed, microglia is considered to function as a double-edged sword since its response is not necessarily neurotoxic, but may be useful to control and clear damage resulting from challenging conditions [42]. In line with these considerations, our results suggest that SERT mutant rats show activated microglia under basal conditions, which may eventually be associated with a chronic inflammatory state, also suggested by increased expression of proinflammatory cytokines. On the other hand, the microglial response to the inflammatory challenge is largely similar in SERT wild-type and mutant rats, suggesting that the intensity of the challenge may not be adequate for discriminating between the two cohorts.

Microglia is maintained in a resting state through neuron-derived signals including CX3CL1 and CD47, which act on their respective receptors CX3CR1 and CD172A expressed by microglia [42,50,51]. However, according to our data, the activation of microglia observed in mutant rats under basal condition cannot be explained by changes in the expression of CX3CL1 and CD47, as their mRNA levels are even higher in the ventral hippocampus of SERT+/− rats. We may speculate that the basal upregulation of these genes may represent a compensatory mechanism aimed at limiting the microglial activation observed in SERT mutant rats under basal conditions. In line with this interpretation, 24 h after the LPS challenge CX3CL1 and CD47 mRNA levels were markedly increased in wild-type animals but not in mutant rats, further suggesting a dysregulation of the mechanisms responsible for the control of microglial function and activation.

Multiple molecular mechanisms may be involved in the differential inflammatory response observed after LPS challenge in SERT mutant rats. For example, it is known that LPS can activate and recruit Toll-like receptors (TLRs), especially TLR4, which appear important in mood-related disorders [52]. However, since hippocampal TLR4 gene expression is not altered in SERT mutant rats (data not shown), other mechanism may be responsible for heightened responsiveness to LPS challenge of SERT^+/−^ rats.

In conclusion, our results demonstrate that the inflammatory/immune system is altered in rats with genetic alterations of the serotonin transporter. Moreover, these animals show a different response to an inflammatory challenge, suggesting that some of the mechanisms that regulate these systems can be compromised, thus rendering the hippocampus more susceptible to the adverse influence of inflammatory mediators. Since depression vulnerability can be associated with increased inflammation, we suggest that the alterations of the immune system observed in animals with a deletion of the SERT gene may contribute to their pathologic phenotype. Furthermore, considering that depressed patients with higher levels of cytokines, as well as individuals carrying the S variant of the 5-HTTLPR polymorphism, are less responsive to antidepressant treatment [53,54], we may speculate that alterations of the immune/inflammatory system in depressed individuals may not only contribute to the pathologic phenotype, but also represent an important component for the response to pharmacological intervention.

## Abbreviations

CX3CL1: Chemokine (C-X3-C Motif) ligand 1 or fractalkine; CX3R1: Chemokine (C-X3-C Motif) receptor 1; CINC: Cytokine-induced neutrophil chemoattractant; DH: Dorsal hippocampus; CD11b: Integrin alpha M or cluster of differentiation; CD47: Integrin-associated protein; IL: Interleukin; L: Long; LPS: Lipopolysaccharide; MIP: Macrophage inflammatory protein; PCR: Polymerase chain reaction; SERT/5-HTT: Serotonin transporter; S: Short; TNF: Tumor necrosis factor; VH: Ventral hippocampus; WT: Wild-type

## Competing interests

The authors declare that they have no competing interests.

## Authors’ contributions

The authors FM, GR, JH, MAR and RM conceived and designed the experiments; the authors FM, FC and CZ performed the experiments and analyzed the data; the authors FM, MAR and RM wrote or contributed to the writing of the manuscript. All the authors have approved the final manuscript.
